# Knockdown of the nucleosome binding protein 1 inhibits the growth and invasion of clear cell renal cell carcinoma cells in vitro and in vivo

**DOI:** 10.1186/1756-9966-31-22

**Published:** 2012-03-15

**Authors:** Shi-Qi Ji, Lin Yao, Xiao-Yu Zhang, Xue-Song Li, Li-Qun Zhou

**Affiliations:** 1 Department of Urology, Peking University First Hospital, Beijing 100034, China; 2 The Institute of Urology, Peking University, Beijing 100034, China; 3 National Urological Cancer Center, Beijing 100034, China; 4 Department of Urology, First Hospital of Peking University, Peking University, No. 8, Xishiku Street, West District, Beijing 100034, China

**Keywords:** Clear cell renal cell carcinoma, NSBP1, Apoptosis, Cell cycle, MMPs

## Abstract

**Background:**

The nucleosome binding protein 1 (HMGN5/NSBP1) is a member of the HMGN protein family and is highly expressed in several kinds of cancer. Nevertheless, the role of NSBP1 in clear cell renal cell carcinoma (ccRCC) remains unclear. This study aimed to confirm the oncogenic role of NSBP1 in ccRCC using in vitro and in vivo models and explore the mechanism by which NSBP1 contributes to ccRCC tumorigenesis.

**Methods:**

NSBP1 expression was detected in renal tissues from 152 ccRCC patients by immunohistochemistry, and examined in ccRCC cell lines by RT-PCR and Western blot analysis. ccRCC cells were transfected by NSBP1 RNAi and cell viability, apoptosis and invasion were detected by cell vitality test, flow cytometry and transwell assay in vitro. Xenograft in nude mice was also employed to examine the tumorigenesis of ccRCC cells depleted of NSBP1.

**Results:**

Immunohistostaining showed strong immunoreactivity of NSBP1 in all ccRCC tissues and NSBP1 expression level was associated with tumor grade (p = 0.04). NSBP1 expression at mRNA and protein levels was high in ccRCC cell lines. Knockdown of NSBP1 induced cell cycle arrest and apoptosis, and inhibited invasion in 786-O cells. Western blot analysis demonstrated increased expression of Bax and decreased expression of Bcl-2, CyclinB1, VEGF, VEGFR-2, MMP-2, MMP-9, c-fos and c-jun in 786-O cells depleted of NSBP1. In vivo study further showed that knockdown of NSBP1 affected the tumorigenesis of ccRCC cells in nude mice.

**Conclusions:**

NSBP1 plays oncogenic role in ccRCCs by promoting cell proliferation and invasion, and could be exploited as a target for ccRCC treatment.

## Introduction

Renal carcinoma is the 13th most common cancer worldwide, with clear cell and clear cell renal cell carcinoma (ccRCC) accounting for most of the renal cell carcinoma (RCC) [1]. Radical nephrectomy is effective to cure early and local ccRCCs, but advanced or metastatic ccRCCs barely respond to chemotherapy or radiotherapy and have poor prognosis. Therefore, it is important to better understand the pathogenesis of aggressive RCC in order to develop effective strategies for the prevention and treatment of RCC.

NSBP1 is a new member of the high mobility group N (HMGN) protein family that modulates the structure and function of chromatin and plays an important role in transcription, histone modifications, DNA replication and DNA repair in living cells[2]. Early study showed that nucleosome binding protein 1 (HMGN5/NSBP1) was abundantly expressed in prostate cancer [3]. In addition, NSBP1 expression was upregulated in squamous cell carcinoma, metastatic MDA-MB-435HM breast cancer cell line and adenocarcinoma, suggesting that NSBP1 may promote tumorigenesis [4-7].

Our previous studies showed that downregulation of NSBP1 expression caused G2 cell cycle arrest, decreased proliferation rate and increased apoptosis rate in prostate cancer cells in vitro [8,9]. Nevertheless, the role of NSBP1 in ccRCC development remains unknown.

Tumor invasion and metastasis are complicated processes, among which proteolytic degradation of extracellular matrix (ECM) and angiogenesis (VEGF) are essential steps. ECM degradation can be promoted by the imbalance between proteolytic proteases and their inhibitors. Extensive studies have shown that matrix metalloproteinases (MMPs) play crucial role in the degradation of ECM to promote tumor invasion and metastasis [10,11].

Therefore, in this study we investigated the role of NSBP1 in ccRCC. First we detected NSBP1 expression in clinical ccRCC tissues and ccRCC cell lines. Then we examined the effects of lentivirus mediated NSBP1 knockdown on the growth and invasion of ccRCC 786-O cells and xenograft tumor growth in nude mice. The results showed that NSBP1 expression was upregulated in ccRCC tissues and ccRCC cell lines, and NSBP1 knockdown could induce apoptosis and inhibit the proliferation and invasion of ccRCC cells, and further decrease ccRCC tumor growth in nude mice.

## Methods

### Clinical samples

A total of 152 patients (aged 52 to 90 years old, median age of 64 years) who underwent surgery from January 2008 to January 2011 in Peking University First Hospital were enrolled in the present study. All patients were of Chinese origin. Paraffin wax-embedded blocks of tumor tissues from each patient were assembled from the archival collections at the Department of Pathology. Survival data of all patients were collected. Among these patients, 20 patients were randomly selected and paired cancer and adjacent tissues were collected from them for Western blot analysis of NSBP1 expression. All adjacent tissues were confirmed to be normal by experienced pathologists. The protocols for the present study were approved by the Ethics Committee of Peking University First Hospital.

### Cell culture

The ccRCC cell lines Caki-2, A498, 786-O and the normal renal tubular epithelial line HK-2 were purchased from American Type Culture Collection (ATCC, Manassas, VA). HK-2 cells were cultured in K-SFM medium (Gibco™ Life Technologies, Grand Island, NY), and other cells were cultured in RPIM-1640 (HyClone, Logan, UT) medium supplemented with 10% Gibco™ FBS (Life Technologies, Grand Island, NY). All cells were cultured at 37°C in a standard humidified incubator containing 5% CO_2 _and 95% O_2_.

### Lentivirus RNAi construct and transfection

The siRNA targeting the human NSBP1 (NM_030763) transcript was designed using the software developed by Ambion (Foster, CA, USA) with the following sequence: PscSI616 CACAGCCTTTCTTTAGCATTTCAAGAGAATGCTAAAGAAAGG-CTGTG/CACAGCCTTTCTTTAGCATTCTCTTGAAATGCTAAAGA-AAGGCTGTG. NSBP1 siRNA or control scramble siRNA was cloned into vector. 786-O cells were seeded onto 6-well plates and grown to 60% confluence on the day of transfection. 4 h before transfection, cells were placed in serum-free media. Cells were transfected with 100 nM siRNA vector diluted in RPMI-1640 according to the manufacturer's protocol. Successful knockdown of NSBP1 was analyzed by Western blot analysis and real-time PCR.

### Immunohistochemistry

Paraffin-embedded tissues were cut into 4 um-thick consecutive sections and were then dewaxed in xylene and rehydrated in graded ethanol solutions. Antigen retrieval was performed following the standard procedure. Sections were cooled and immersed in a 0.3% hydrogen peroxide solution for 15 min to block endogenous peroxidase activity, and then rinsed in PBS for 5 min. Non-specific labeling was blocked by incubation with 5% bovine serum albumin at room temperature for 30 min. Sections were then incubated with primary rabbit anti-human antibody against NSBP1 (diluted in 1:100, Abcam, ab56031, Cambridge, MA) at 4°C overnight, rinsed with PBST, incubated with horseradish peroxidase-conjugated Santa Cruz™ goat anti-rabbit IgG secondary antibody (Santa Cruz, CA), developed by peroxidase-conjugated streptavidin and DAB, and counterstained by hematoxylin. All slides were examined independently by two pathologists, who were not informed about patients' clinical data. Specimens were then grouped according to stage (T1-T4) and specific staining intensity. The staining intensity was scored as "-" for negative, "+" for moderate, and "++" for strong staining.

### Quantitative real-time PCR assay

Total RNA was extracted from the cells using Trizol (Invitrogen) according to the manufacturer's protocol. First-strand cDNA was generated using 2 μg total RNA via MMLV-reverse transcriptase using High Capacity RNA-to-cDNA kit (Promega) with random primers. A final reaction of 20 ul was used to determine the mRNA level by real-time PCR using an ABI Prism 7300 (Applied Biosystems, Foster City, CA, USA). The specific primers were as follows: NSBP1, 5'-TCGGCTTTTTTTCTGCTGACTAA-3'(forward) and 5'-CTCTTTGGCTCCTGCCTCAT-3'(reverse); Actin, 5'-GTGGACATCCGCAAAGAC3'(forward) and 5'-ATCAACGCAATGTGGGAAA-3'(reverse). Thermal cycling was initiated with a denaturation step for 5 min at 94°C followed by 36 cycles done in three steps: 30 s at 94°C, 30 s at 58°C and 1 min at 72°C.

### Cell proliferation assay

Cell proliferation was assessed using the CellTiter 96 Aqueous assay kit (Promega, Madison, WI). After transfection, the cells (10,000/well) were seeded in 96-well plates and incubated at 37°C, and cell proliferation was assessed after 96 h based on the absorbance measured at 570 nm using a multiwell spectrophotometer.

### Flow cytometry

Apoptosis was evaluated by Annexin V-PE/7-AAD staining followed by flow cytometry analysis. After cells were plated in 6-well plates at a density of 1 × 10^5^/well and cultured at 37°C in 5% CO2 incubator for three days, they were transfected with NSBP1 siRNA or scramble siRNA vector, the cells were gently trypsinized and washed with ice-cold PBS after 72 h. At least 20,000 cells were resuspended in 500 μL 1 × binding buffer, stained with 5 μL 7-AAD (25 μg mL^-1^) and 1 μL Annexin V-PE and immediately analyzed with a FACScalibur flow cytometer (Becton Dickinson, Erembodegem, Belgium).

### Western blot analysis

inhibitors. Protein samples(40 ug)were separated in 10% SDS-polyacrylamide gels and transferred to PVDF membranes. The membranes were blocked with nonfat milk in TBST, and probed with primary antibodies CyclinB1 (CST-4138), CyclinD1 (CST-2978), Proliferating Cell Nuclear Antigen (PCNA, CST-2586), Bax (CST-2772), Bcl-2 (CST-2876), VEGF (CST-2445), VEGFR-2 (CST-2472), MMP-2 (CST-4022), MMP-9 (CST-3852) (CST indicated Cell Signaling Technology, Beverly, MA, USA). c-fos (santa cruz-52), c-jun (santa cruz-1694), GAPDH (santa cruz-137179), or β-Actin (santa cruz-81178), and secondary antibodies goat anti-mouse IgG (santa cruz), goat anti-rabbit IgG (santa cruz) (santa cruz indicated Santa Cruz Biotech, Santa Cruz, CA, USA). Immunoreactivity signals were developed using ECL kit (GE Healthcare Bioscience, Piscataway, NJ, USA). protease and phosphatase with Whole-cell extracts were prepared in RIPA buffer

### Cell invasion assay

Cell invasion assay was performed with 24-well Transwell insert (pore size 8 μm, Corning, NY). After transfection, 786-O cells were starved in serum free medium overnight, and 3-5 × 10^4 ^cells were resuspended in 200 ul serum-free medium and placed in the upper chambers with 8 μm filter pores in triplicate. The membrane undersurface was coated with 30 ul ECM gel from Engelbreth-Holm-Swarm mouse sarcoma (BD Biosciences, Bedford, MA, USA) mixed with RPMI-1640 serum free medium in 1:5 dilution for 30 min at 37°. The lower chamber was filled with 500 ul 10% FBS as the chemoattractant and incubated for 48 h. At the end of the experiments, the cells on the upper surface of the membrane were removed by cotton buds, and the cells on the lower surfaPBS-buffered paraformaldehyde and stained with 0.1% crystal violet. Five visual fields were chosen randomly for each insert and photographed under a light microscope at 200 × magnification. The cells were counted and the data were summarized by means ± standard deviation and presented by a percentage of controls. ce of the insert were fixed in 4%.

### Gelatin zymography assay

After transfection, the cells were cultured in serum free medium for 24 h. Then the medium was collected by centrifugation at 4,000 rpm for 15 min at 4°C, and subjected to zymographic SDS-PAGE containing 0.1% gelatin (w/v). The gels were washed and incubated in incubation buffer for 48 h, then stained with Coomassie Brilliant Blue and destained. The zones of gelatinolytic activity were shown by negative staining.

### Tumourigenesis assay in nude mice

Female BALB/c^nu/nu^mice (4-6 weeks old, weighed 25-30 g) were maintained in a germ-free environment in the animal facility. NSBP1 knockdown or control 786-O cells were cultured in 100-mm dishes and trypsinized. The cells (10 ^6 ^in 100 ul medium) were infused subcutaneously in the armpit area. Tumor diameter was measured every 5 days, and tumor volume was calculated by length × width^2^× 0.5. Mice were sacrificed after 1.5 months.

### Statistical analysis

Values were represented as mean ± SD for at least triplicate determination, and analyzed using Fisher's exact test and Kruskal-Wallis test. All statistical analyses were performed using SPSS 13.0 and P < 0.05 was considered as statistically significant.

## Results

### NSBP1 expression is high in ccRCC tissues

**Figure 1:**
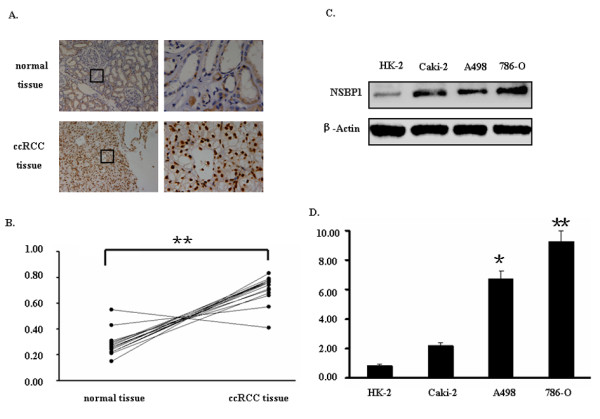
**NSBP1 expression is high in ccRCC tissues and cells**. (A), Representative immunohistochemistry staining of NSBP1 in control renal tissue and ccRCC tissue. NSBP1 immunoreactivity in brown was predominantly localized in the nucleus. Original magnification: X10 (a, c), X40 (b, d). (B) Ratio between protein expression levels of NSBP1 and β-Actin in pairs of ccRCC and normal tissue from 20 patients was calculated based on Western blot analysis. (C), Western blots demonstrating the expression of NSBP1 in different ccRCC cells. Actin served as loading control. (D), Real-time PCR assay showing the relative NSBP1 mRNA level in different ccRCC cells. *p < 0.05, **p < 0.01, versus HK-2 cells.

We examined NSBP1 expression in ccRCC tissue by immunohistochemistry. As shown in Figure 1A, NSBP1 staining was weak in the normal renal tissues but strong in ccRCC tissues. Western blot analysis of 20 paired adjacent normal renal tissues and ccRCC tissues confirmed the high expression of NSBP1 in ccRCC tissues (p = 0.006) (Figure 1B). Most importantly, we found that NSBP1 staining intensity was correlated with the clinical and pathologic characteristics of ccRCC (Table 1). NSBP1 expression was positively correlated with the tumor grade and pathologic stage.

**Table 1:** Correlation of NSBP1 expression with clinical and pathological characteristics of renal carcinoma

Characteristics	Cases	NSBP1 immunoreactivity						P
		-		+		++		
		Cases (%)		Cases (%)		Cases (%)		
**Gender**								0.653
**Male**	129	18	11.8	33	21.7	78	51.3	
**Female**	23	3	2.0	8	5.3	12	7.9	
								
**Age (years)**	60.4 ± 8.9	59.8 ± 9.7		60.2 ± 9.8		61.3 ± 11		
**Grade**								0.040*
1	0	0		0		0		
2	63	14	9.2	16	10.5	33	21.7	
3	89	7	4.6	25	16.5	57	37.5	
								
**Pathologic stage**								0.002**
T1	18	10	6.6	5	3.3	3	2.0	
T2	35	6	3.9	12	7.9	17	11.1	
T3	47	3	2.0	15	9.9	29	19.1	
T4	52	2	1.3	9	5.9	41	27.0	

Correlation significant at the 0.05 level (one-tailed)

*P < 0.05; **P < 0.01

### NSBP1 expression is high in ccRCC cells

We examined NSBP1 expression in ccRCC cell lines and the normal renal tubular epithelial line cells by quantitative real-time RT-PCR and Western blot. NSBP1 protein level was higher in ccRCC cell lines than normal renal tubular epithelial line cells (Figure 1C). Similarly, NSBP1 mRNA level was increased in ccRCC cell lines compared to normal renal tubular epithelial line cells (Figure 1D).

### NSBP1 knockdown decreases the proliferation of ccRCC cells

**Figure 2:**
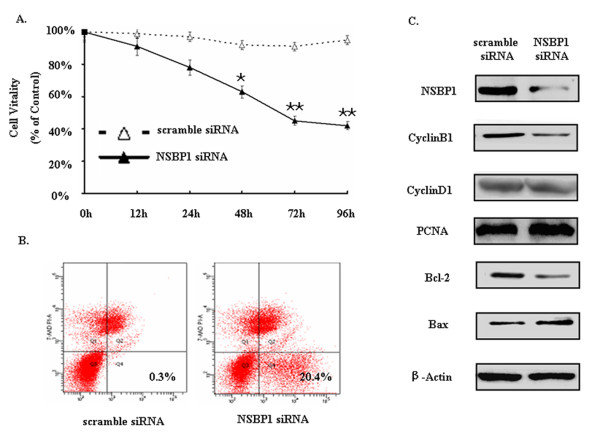
**NSBP1 knockdown decreases the proliferation of ccRCC cells**. (A), MTT assay showing that NSBP1 knockdown significantly reduced the proliferation of ccRCC cells over the 96 h period. (B), Annexin V-PE/7-AAD staining in FCM assays showing the ratio of apoptosis in different 786-O cells. (Data were presented as mean ± SEM, n = 3). (C), Representative blots demonstrating the reduced protein level of NSBP1 in NSBP1 siRNA transfected 786-O cells compared to scramble siRNA transfected cells. In addition, Bax protein level was increased and CyclinB1 and Bcl-2 levels were decreased in NSBP1 siRNA transfected 786-O cells compared to scramble siRNA transfected cells.

To investigate the role of NSBP1 in the proliferation of ccRCC cells, we employed the loss of function approach. 786-O cells were transfected with NSBP1 siRNA or scramble siRNA as control and cell proliferation was evaluated by MTT assay. The results showed that NSBP1 knockdown significantly reduced proliferation of ccRCC cells over the 72 h period (Figure 2A). Lentivirus short hairpin constructs against NSBP1 (PscSI616) was efficient and specific in the knockdown of NSBP1 in 786-O cells and the inhibitory efficiency at protein level was 74.8 ± 2.1% based on Western blot analysis (Figure 2C).

The apoptosis of ccRCC cells was examined by FCM after the cells were transfected with NSBP1 siRNA or scramble siRNA as control. The apoptotic ratio was increased in NSBP1 knockdown 786-O cells compared to control (Figure 2B). To confirm that NSBP1 knockdown could inhibit proliferation and induce apoptosis in ccRCC cells, we examined the expression of apoptosis and cell cycle related proteins and found that Bax protein level was significantly increased while CyclinB1 and Bcl-2 protein levels were decreased in NSBP1 knockdown cells compared with control (Figure 2C). These data provide evidence that NSBP1 modulates cell cycle and antagonizes apoptosis to promote the oncogenic potential of ccRCC cells.

### NSBP1 knockdown inhibits the invasion of ccRCC cells

**Figure 3:**
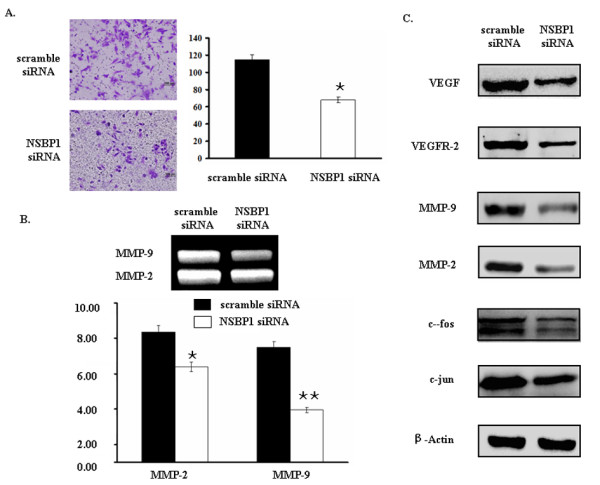
**NSBP1 knockdown inhibits the invasion of ccRCC cells**. (A), Representative photos showing the invasion of ccRCC cells into the lower chamber of transwell. ×200. (B), Gelatin zymography assay showing that MMP-9 and MMP-2 activities were decreased in NSBP1 knockdown 786-O cells. Data shown were mean ± SEM from three independent experiments. (C), Western blot analysis showing that the expression of VEGF, VEGFR-2, MMP-2, MMP-9, c-fos and c-jun were significantly decreased in NSBP1 knockdown 786-O cells. Data shown were mean ± SEM from three independent experiments. *p < 0.05, **p < 0.01, versus the scramble siRNA transfected control group.

Next we assessed the role of NSBP1 in cell invasion, an important aspect of ccRCC metastasis. By transwell assay we found that NSBP1 knockdown cells showed few number of invading cells compared to control group which expressed high level of NSBP1 (Figure 3A). The number of cells crossing the matrigel was 62.3 ± 3.1 in NSBP1 siRNA group versus 110.7 ± 3.1 in scramble siRNA control group (P < 0.05). Moreover, gelatin zymography assay demonstrated that NSBP1 knockdown efficiently decreased MMP-2 and MMP-9 enzymatic activity, especially MMP-9 enzymatic activity (Figure 3B). To address whether decreased MMP-9 and MMP-2 activity is due to the downreguation of their expression after NSBP1 knockdown, we examined the expression of MMP-9, MMP-2 and their upstream transcription factors c-fos and c-jun. Western blot analysis demonstrated that NSBP1 knockdown downregulated the expression of VEGF, VEGFR-2, MMP-2, MMP-9, c-fos and c-jun (Figure 3C). Taken together, these data suggest that NSBP1 upregulates the expression of MMP-2 and MMP-9 via c-fos and c-jun. The increased MMPs activity and angiogenesis then contributes to the migration and invasion of ccRCC cells.

### NSBP1 knockdown inhibits ccRCC growth in xenograft nude mice

**Figure 4:**
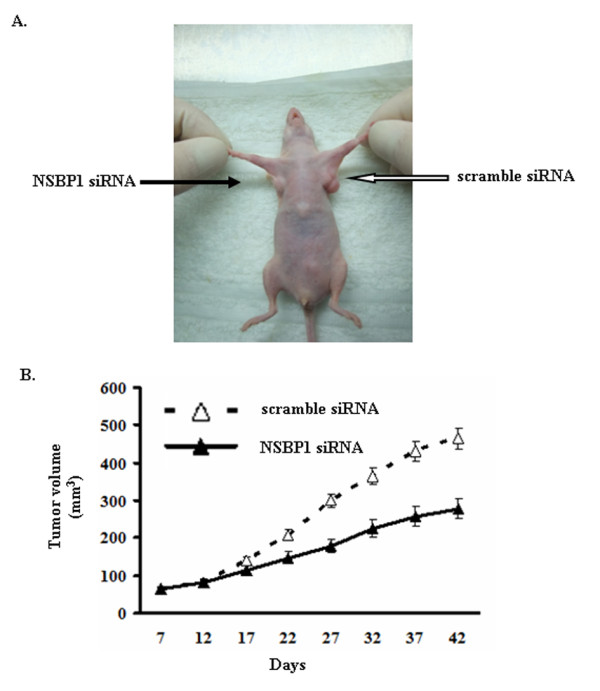
**NSBP1 knockdown inhibits the tumorigenicity of ccRCC cells in vivo**. (A), A representative nude mice showing the different morphology of the tumors derived from NSBP1 siRNA transfected 786-O cells (left side) and scramble siRNA transfected control cells (right side). (B), the growth curve of the tumors (n = 10).

To further investigate the role of NSBP1 in ccRCC in vivo, we established xenograft ccRCC by subcutaneous injection of 1 × 10^6 ^NSBP1 knockdown 786-O cells or the corresponding scramble siRNA transfected control cells into the flanks of BALB/c nude mice (n = 10). After 6 weeks, we observed that the volume of tumor derived from NSBP1 knockdown cells was significantly smaller than that derived from control cells (Figure 4). These data demonstrate that NSBP1 knockdown inhibits the tumorigenicity of ccRCC cells in vivo.

## Discussion

NSBP1 was identified as a new member of the HMGN protein family in 2001 [12,13]. As a nuclear protein, NSBP1 modulates the structure and function of chromatin and plays an important role in transcription, DNA replication and repair [14-16]. Interestingly, recent studies demonstrated that NSBP1 expression was abnormally high in a variety of solid tumors, indicating the oncogenic role of NSBP1 [4-7],

In this study, we found that NSBP1 expression was significantly higher in ccRCC tissues and cell lines than normal renal tissue and cell lines. These data suggest that NSBP1 overexpression is correlated with the progression of ccRCC.

To elucidate the role of NSBP1 in the tumorigenesis of ccRCC, we employed loss of function approach via the knockdown of endogenous NSBP1 expression in ccRCC cells. Notably, we found that NSBP1 knockdown inhibited the proliferation rate of ccRCC cells through MTT assay. Furthermore, our experiments showed that knockdown of NSBP1 led to increased Bax expression and decreased CyclinB1, Bcl-2 expression. These results suggest that downregulation of NSBP1 expression causeds G2 cell cycle arrest, decreases the proliferation rate and increases apoptosis rate in ccRCC cells in vitro[17-20].

Metastasis is an important aspect of ccRCC. To characterize the role of NSBP1 in ccRCC metastasis, we employed in vitro invasion assay and found that NSBP1 knockdown led to decreased invasion of ccRCC cells. Tumor invasion and metastasis are crucially dependent on MMPs and VEGF [10,11,20]. MMP-2 and MMP-9 play important roles in the degradation of the ECM, including type IV collagen, and their activity and expression are correlated with metastatic abilities and prognosis of cancer[21,22]. Our results showed that silencing of NSBP1 in 786-O cells decreased MMP-2 and MMP-9 activity based on zymography assay. In addition, we found that MMP-2 and MMP-9 expression as well as the expression of transcription factors c-fos and c-jun were decreased in NSBP1 knockdown cells. These data suggest that NSBP1 modulates the expression of MMPs and VEGF/VEGFR-2 thus influencing the invasion behavior of ccRCC cells.

Finally, to demonstrate that NSBP1 contributes to ccRCC development in vivo, we employed xenograft nude mice model and found that NSBP1 knockdown suppressed tumor growth of ccRCC cells, indicating that NSBP1 promotes the tumorigenicity of ccRCC cells in vivo.

In summary, here we present both in vitro and in vivo evidence that NSBP1 promotes ccRCC cells growth and invasion. NSBP1 plays important role in the regulation of apoptosis and invasion of ccRCC cells by regulating the expression of Bcl-2, Bax, CyclinB1 VEGF/VEGFR-2 and MMPs. Based on these findings, intervention with NSBP1 expression may provide a therapeutic approach in ccRCC development and metastasis.

## Competing interests

The authors declare that they have no competing interests.

## Authors' contributions

SQJ supervised research project, participated in the data collection, drafted the manuscript. LY participated in the data collection, supervised ICH. XYZ participated in the data collection. XSL carried out the operation. LQZ carried out the operation, acted as corresponding author and did the revisions. All authors read and approved the final manuscript.

## References

[B1] Ljungberg B Campbell SC Choi HY Jacqmin D Lee JE Weikert S Kiemeney LA The epidemiology of renal cell carcinoma Eur Urol 2011 60 615 621 10.1016/j.eururo.2011.06.049 21741761

[B2] Hock R Furusawa T Ueda T Bustin M HMG chromosomal proteins in development and disease Trends Cell Biol 2007 17 72 79 10.1016/j.tcb.2006.12.001 17169561 PMC2442274

[B3] Wang JW Zhou LQ Yang XZ Ai JK Xin DQ Na YQ Guo YL The NSBP1 expression is up-regulated in prostate cancer cell Basic Med Sci Clin 2004 24 393 397

[B4] Huang C Zhou LQ Song G Effect of nucleosomal binding protein 1 in androgen-independent prostatic carcinoma Zhong hua Yi Xue Za Zhi 2008 88 657 660 18642763

[B5] Green J Ikram M Vyas J Patel N Proby CM Ghali L Leigh IM O'Toole EA Storey A Overexpression of the Axl tyrosine kinase receptor in cutaneous SCC-derived cell lines and tumours Br J Cancer 2006 94 1446 1451 10.1038/sj.bjc.6603135 16641895 PMC2361292

[B6] Li DQ Hou YF Wu J Chen Y Lu JS Di GH Ou ZL Shen ZZ Ding J Shao ZM Gene expression profile analysis of an isogenic tumour metastasis model reveals a functional role for oncogene AF1Q in breast cancer metastasis Eur J Cancer 2006 42 3274 3286 10.1016/j.ejca.2006.07.008 16979889

[B7] Tang WY Newbold R Mardilovich K Jefferson W Cheng RY Medvedovic M Ho SM Persistent hypomethylation in the promoter of nucleosomal binding protein1 (Nsbp1) correlates with overexpression of Nsbp1 in mouse uteri neonatally exposed to diethylstilbestrol or genistein Endocrinology 2008 149 5922 5931 10.1210/en.2008-0682 18669593 PMC2613067

[B8] Zhou LQ Song G He ZS Hao JR Na YQ Effect of inhibiting nucleosomal binding protein 1 on proliferation of human prostate cancer cell line LNCaP Chin Med J 2007 86 404 408 17456383

[B9] Jiang N Zhou LQ Zhang XY Downregulation of the nucleosome-binding protein 1 (NSBP1) gene can inhibit the in vitro and in vivo proliferation of prostate cancer cells Asian J Androl 2010 12 709 717 10.1038/aja.2010.39 20531280 PMC3739312

[B10] Mukherjee S Roth MJ Dawsey SM Yan W Rodriguez-Canales J Erickson HS Hu N Goldstein AM Taylor PR Richardson AM Tangrea MA Chuaqui RF Emmert-Buck MR Increased matrix metalloproteinase activation in esophageal squamous cell carcinoma J Transl Med 2010 8 91 10.1186/1479-5876-8-91 20920372 PMC2958908

[B11] Rak J Milsom C May L Klement P Yu J Tissue factor in cancer and angiogenesis: the molecular link between genetic tumor progression, tumor neovascularization, and cancer coagulopathy Semin Thromb Hemost 2006 32 54 70 Review 10.1055/s-2006-933341 16479463

[B12] Rochman M Malicet C Bustin M HMGN5/NSBP1: A new member of the HMGN protein family that affects chromatin structure and function Biochim Biophys Acta 2010 1799 86 92 20123071 10.1016/j.bbagrm.2009.09.012 PMC2818475

[B13] Shirakawa H Herrera JE Bustin M Postnikov Y Targeting of high mobility group-14/-17 proteins in chromatin is independent of DNA sequence J Biol Chem 2000 275 37937 37944 10.1074/jbc.M000989200 10973947

[B14] Catez F Lim JH Hock R Postnikov YV Bustin M HMGN dynamics and chromatin function Biochem Cell Biol 2003 81 113 122 10.1139/o03-040 12897844

[B15] Rochman M Postnikov Y Correll S Malicet C Wincovitch S Karpova TS McNally JG Wu X Bubunenko NA Grigoryev S Bustin M The interaction of NSBP1/HMGN5 with nucleosomes in euchromatin counteracts linker histone-mediated chromatin compaction and modulates transcription, Mol Cell 2009 35 642 656 10.1016/j.molcel.2009.07.002 PMC2757144 19748358

[B16] Rattner BP Yusufzai T Kadonaga JT HMGN proteins act in opposition to ATP-dependent chromatin remodeling factors to restrict nucleosome mobility Mol Cell 2009 34 620 626 10.1016/j.molcel.2009.04.014 19524541 PMC2709789

[B17] Rozenblat S Grossman S Bergman M Gottlieb H Cohen Y Dovrat S Induction of G2/M arrest and apoptosis by sesquiterpene lactones in human melanoma cell lines Biochem Pharmacol 2008 75 369 382 10.1016/j.bcp.2007.08.024 17919456

[B18] Beauman SR Campos B Kaetzel MA Dedmana JR CyclinB1 expression is elevated and mitosis is delayed in HeLa cells expressing autonomous CaMKII Cell Signal 2003 15 1049 1057 10.1016/S0898-6568(03)00068-8 14499348

[B19] Chulu Julius LC Huang Wei R Wang L Shih Wen L Liu Hung J Avian Reovirus Nonstructural Protein p17-Induced G2/M Cell Cycle Arrest and Host Cellular Protein Translation Shutoff Involve Activation of p53-Dependent Pathways J Virol 2010 84 7683 7694 10.1128/JVI.02604-09 20484520 PMC2897625

[B20] Yin J Chen G Liu Y Liu S Wang P Wan Y Wang X Zhu J Gao H Downregulation of SPARC expression decreases gastric cancer cellular invasion and survival J Exp Clin Cancer Res 2010 29 59 10.1186/1756-9966-29-59 20525171 PMC2892439

[B21] Rink M Chun FK Robinson B Sun M Karakiewicz PI Bensalah K Fisch M Scherr DS Lee RK Margulis V Shariat SF Tissue-based molecular markers for renal cell carcinoma Minerva Urol Nefrol 2011 63 293 308 21996985

[B22] Chang HR Chen PN Yang SF Sun YS Wu SW Hung TW Lian JD Chu SC Hsieh YS Silibinin inhibits the invasion and migration of renal carcinoma 786-O cells in vitro, inhibits the growth of xenografts in vivo and enhances chemosensitivity to 5-fluorouracil and paclitaxel Mol Carcinog 2011 50 811 823 10.1002/mc.20756 21574189

